# Tunnel Crack Detection Method and Crack Image Processing Algorithm Based on Improved Retinex and Deep Learning

**DOI:** 10.3390/s23229140

**Published:** 2023-11-13

**Authors:** Jie Wu, Xiaoqian Zhang

**Affiliations:** 1 School of Defense, Xi’an Technological University, Xi’an 710021, China; 2 School of Electronic and Information Engineering, Xi’an Technological University, Xi’an 710021, China; zhangxiaoqian@xatu.edu.cn

**Keywords:** tunnel cracks, multi-scale Retinex decomposition, deep learning, crack segmentation

## Abstract

Tunnel cracks are the main factors that cause damage and collapse of tunnel structures. How to detect tunnel cracks efficiently and avoid safety accidents caused by tunnel cracks effectively is a research hotspot at present. In order to meet the need for efficient detection of tunnel cracks, the tunnel crack detection method based on improved Retinex and deep learning is proposed in this paper. The tunnel crack images collected by optical imaging equipment are used to improve the contrast information of tunnel crack images using the image enhancement algorithm, and this image enhancement algorithm has the function of multi-scale Retinex decomposition with improved central filtering. An improved VGG19 network model is constructed to achieve efficient segmentation of tunnel crack images through deep learning methods and then form the segmented binary image. The Zhang–Suen fast parallel-thinning method is used to obtain the skeleton map of the single-layer pixel, and the length and width information of the tunnel cracks are obtained. The feasibility and effectiveness of the proposed method are verified by experiments. Compared with other methods in the literature, the maximum deviation in the length of the tunnel crack is about 5 mm, and the maximum deviation in the width of the tunnel crack is about 0.8 mm. The experimental results show that the proposed method has a shorter detection time and higher detection accuracy. The research results of this paper can provide a strong basis for the health evaluation of tunnels.

## 1. Introduction

Tunnel detection is the basic means to ensure its safety performance; the detection result is an important index to measure the safety of the tunnel, and it is also a research hotspot in the current tunnel construction. The cracks generated in the tunnel are the core factors affecting the safety of the tunnel. At the same time, the cracks generated in the tunnel will destroy the overall structure of the current tunnel; this result affects the safety and stability of the tunnel structure. Because the existence of tunnel cracks is an important source of tunnel disasters, the traditional tunnel crack detection method mainly relies on manual inspection, which is not only time-consuming and has high labor costs but also has some problems, such as missed detection and false detection. It has been unable to meet the rapid growth of the number and length of tunnel construction in China in terms of efficiency, accuracy, and safety. 

With the development of technology, especially the development of visual imaging technology, tunnel non-destructive testing technology based on intelligent technology such as optical, electrical, and image detection is developing rapidly. Laser scanning technology and photography technology are the main detection technologies at present. Han et al. [1] used wavelet transform to process each scanning line in the point-cloud data collected by the three-dimensional laser scanner, which can detect and extract the point-cloud data containing crack information well, thus reducing the workload and improving the work efficiency. According to the characteristics of 3D laser scanning data of surface cracks in mining subsidence areas, Li et al. [2] applied 3D laser scanning technology to detect surface cracks in mining subsidence areas and used wavelet analysis to identify the position of surface cracks according to the characteristics of 3D laser scanning data. In order to improve the shortcomings of traditional monitoring methods for goaf, Chen et al. [3] proposed a point-cloud denoising method based on a KD Tree and a registration method based on point-cloud characteristics and obtained realistic 3D morphological data of goaf through multiple scanning by a 3D laser scanner. Liu et al. [4] studied a new method based on ground laser scanning and image processing technology to extract highway crack information in mining areas. The detection results of this method are high detection accuracy, which solves the problem that highway cracks affect normal operation. Ground photogrammetry has also been used for structural deformation monitoring, especially for three-dimensional geometric evaluation and displacement monitoring of concrete pavements and masonry bridges. It can also quickly and accurately extract the width and length information of cracks in tunnel crack images. 

The research on tunnel cracks by visual method has achieved corresponding results, which has laid a certain foundation for the development of current tunnel crack detection technology. The tunnel-lining crack detection method based on machine vision needs to configure the light source to supplement the light of the camera and has higher requirements for synchronous shooting, high-speed storage, and high-performance recognition ability of image features [5,6,7]. Li et al. [8] took the lining-crack image of a subway shield tunnel as the research object, quantified the crack disease by digital image processing, obtained the length and width parameters of the crack by the dynamic block method, and established the tunnel crack disease grade and classification standard by the K-means clustering algorithm and partial least squares regression method. Aiming at the problems of insufficient exposure, uneven illumination, and serious noise of the collected tunnel-lining images, Tang et al. [9] adopted bilateral filtering for denoising and processing, segmented the images based on image adaptive segmentation combined with the segmentation algorithm of threshold and edge information, and obtained the binarized image of the tunnel-lining images. The real length and width of the crack are calculated by the camera dimensioning method. Wang et al. [10] proposed an inflection point identification method for tunnel-lining fracture skeleton. By using the improved eight-directional Freeman chain code technology, suspicious inflection points were initially identified, and false inflection points were removed to obtain the real inflection point information. According to the brightness difference in different directions of the lining-crack image, Wang et al. [11] realized crack recognition by optimizing the segmentation of crack pixels and background pixels in the lining-crack image according to the brightness differences in different directions of the lining-crack image. Stent et al. [12] developed a crack identification method based on multiple linear CCD cameras and light source devices, which can identify cracks with a width greater than 0.3 mm. Xie et al. [13] proposed a fast crack extraction method combining target recognition and semantic segmentation. The faster R-CNN network is used to identify the target of the original lining image, and then the U-Net semantic segmentation network is used to segment the cracks at the pixel level. The experimental results show that this method can significantly improve the speed and accuracy of crack identification. 

Hao et al. [14] proposed a YOLOv4 highway pavement crack detection method that introduces a Ghost module and ECA. By introducing the Ghost module in lightweight GhostNet to optimize the backbone feature extraction network of YOLOv4, the lightweight model YOLOV4-Light was established. The high-efficiency channel attention (ECA) mechanism was added to the prediction output of the model to enhance the ability to extract fracture features. Li et al. [15] proposed a deep bridge crack classification (DBCC) model based on convolutional neural networks (CNNs) and combined it with an improved window sliding algorithm to detect the cracks in the bridge. Compared with the traditional detection algorithm, this algorithm has better recognition effect and stronger generalization ability. In order to improve the detection reliability of the tunnel-lining crack detection platform and the accuracy of image recognition, Wang et al. [16] proposed a tunnel-lining crack detection method based on image compensation. The feature points of enhanced images were extracted, an adaptive motion estimation model matching the feature points was established, and a Kalman filtering algorithm and bicubic interpolation were used to compensate for the images collected during the detection platform movement. The cracks segmentation method of lining images is studied by combining image adaptive block filtering and morphology. The results show that the proposed method not only compensates for the errors existing in the detection platform movement but also can detect the cracks of tunnel lining with high precision.

In addition, Xue et al. [17] used a convolutional neural network (CNN) to train samples and establish a classification system for tunnel-lining feature images. Based on the CNN model GoogLeNet, an optimized convolution kernel was adopted, and the inception module and network structure were improved, which can have better robustness for image processing under complex background conditions. Marco Domaneschim [18] et al. conducted a four-point bending test on reinforced concrete beams until the steel bars were bent and evaluated their structure and damage. Different non-destructive testing (NDT) techniques are used to monitor the deterioration process, and acoustic emission (AE) sensors attached to the material surface are used to monitor the initiation and expansion of cracks, providing strong support for the realization of a reliable real-time structural alarm system. Chen Jia et al. [19] proposed a shallow, deep convolutional neural network model based on VGG to classify pavement damage images, and the classification and recognition accuracy of pavement damage images reached more than 98%. He [20] proposed an automatic measurement method based on machine vision, in which a U-Net convolutional neural network was used to segment the image, and laser ranging was used to measure the image scale, which improved the efficiency and accuracy of crack measurement and met the practical application requirements.

The description of the above detection methods mainly focuses on the detection technologies based on laser scanning and photography. The processing and registration process of the point-cloud data of the crack information obtained by three-dimensional laser scanning is very complicated. Meanwhile, the resolution of laser scanning is limited by the laser beam and sensor, and very small details cannot be captured. It will cause a fuzzy phenomenon at the end of the tunnel crack, resulting in inaccurate crack detection results. In addition, for the detection technology of photography, the image accuracy of the typical linear-array CCD scanning cracks is easily affected by the scanning motion accuracy, which affects the detection accuracy. Although the light source device is added when collecting the original crack images, some images still have uneven illumination, which will cause the loss of crack details. This results in large errors in crack detection results. Therefore, in order to accurately detect cracks, tunnel crack images must be clear. In this paper, a surface-array CCD camera equipped with a light source device is used to collect tunnel cracks, and multi-scale Retinex decomposition with improved bilateral filtering is adopted to enhance the image processing, which effectively improves the contrast of the image and reduces the distortion of the image. Furthermore, the detailed information on the crack edge is enhanced, and an improved VGG19 network model is constructed to achieve accurate and efficient segmentation of tunnel cracks. Finally, the crack information is quantitatively characterized, and the length and width information of cracks are accurately obtained, providing strong data support for the evaluation of the severity of crack diseases. The tunnel crack detection method studied in this paper, the main work and contributions are as follows:

(1) Aiming at the problem that the complex illumination environment inside the tunnel leads to the shadow of the tunnel crack image, the improved bilateral filtering is combined with the multi-scale Retinex decomposition to output the illumination image and the reflection image, and the corresponding mean image is obtained by γ correction. Finally, the enhanced image is output to highlight the unclear texture information of the tunnel crack image and improve the contrast of the tunnel crack image.

(2) The advantages and disadvantages of tunnel crack identification are restricted by the reliability of the whole detection system. Especially on the premise of a small sample dataset, how to effectively improve the accuracy of tunnel crack identification is a crucial and key issue at present. In order to improve the accuracy of tunnel crack detection and improve the robustness of the network model, when the tunnel image is collected, a large sample of the whole tunnel is collected, and the tunnel image is expanded before the image segmentation, and the image segmentation is realized efficiently by the improved VGG19 network model.

(3) According to the extracted tunnel crack information, scan along the crack skeleton trend and introduce Freeman chain code to calculate its length. In the binary image of the crack after processing, the range of the edges on both sides of the crack is determined along the normal vector of the crack, which is the local width of the crack, and the maximum width of the crack is selected as the real width of the crack.

The remainder of this paper is organized as follows: Section 2 states the principle and method of tunnel crack detection. Section 3 states the image enhancement algorithm of multi-scale Retinex decomposition based on improved central filtering. Section 4 states the crack segmentation method based on the improved VGG19 network model. Section 5 states the quantitative characterization of cracks. Section 6 gives the experimental and analysis. Finally, Section 7 provides a summary of this paper.

## 2. Principle and Method of Tunnel Crack Detection

### 2.1. Principle of Tunnel Crack Detection

**Figure 1:**
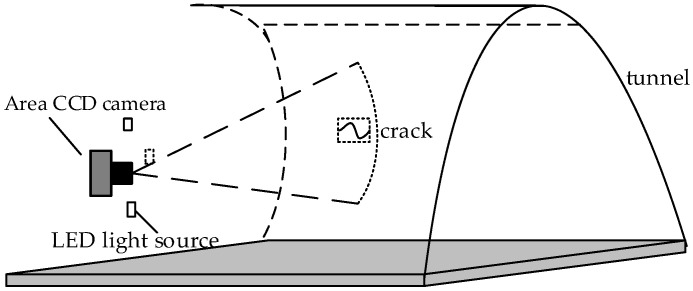
Principle diagram of tunnel crack detection.

When collecting two-dimensional images of tunnel cracks, linear-array cameras are usually used as a tool for collecting images due to their small image distortion, large dynamic range, and non-tailing of images collected under high-speed motion. This acquisition system uses an area-array CCD camera and a high-definition industrial lens to collect tunnel crack images. Due to the lack of lighting equipment in the tunnel, the environment in the tunnel is poor, and the lighting conditions are harsh. So, it is necessary to ensure that the light source has a certain beam width when the image is collected. According to the spectral analysis and light source irradiation model, three rows of high-brightness LED light sources are configured around the lens of the area-array CCD camera. The beam width is more than 20 cm, the brightness is high, and the uniformity is good. In this way, sufficient light intensity and good light-intensity uniformity can be guaranteed to obtain better tunnel images. The principle of tunnel crack detection is shown in Figure 1.

### 2.2. Tunnel Crack Image Detection Method

**Figure 2:**
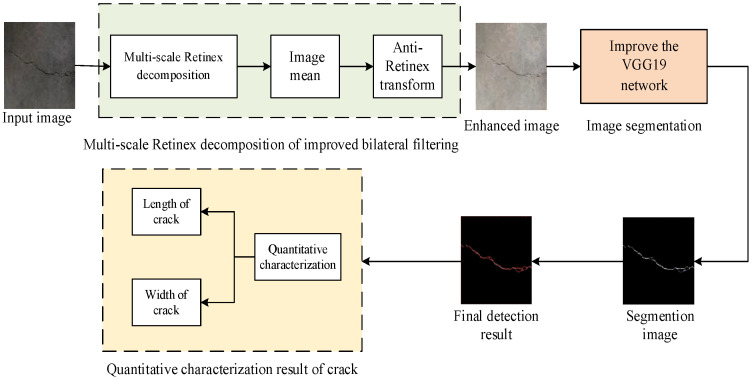
Tunnel crack image processing flowchart.

Due to the influence of insufficient light and uneven lighting inside the tunnel, the image part collected by the detection system has the characteristics of low contrast, resulting in incomplete or inaccurate crack information extracted, which is not conducive to the accurate evaluation of tunnel diseases. In order to improve the brightness and contrast of the image, fully display the crack information in the image, and the bilateral filtering has the ability of anisotropy and edge preservation [21,22], the multi-scale Retinex decomposition of the improved bilateral filtering is used to enhance the image. The improved bilateral filtering is used as the center of Retinex to decompose the image, which effectively improves the contrast of the image and reduces the distortion of the image because the bilateral filtering selects the appropriate filtering neighborhood size, which can not only maintain the edge and texture results of the image but also have a better smoothing effect. The enhanced tunnel image is used as the input information of the improved VGG19 network model, and the deep learning method is used to realize the efficient segmentation of the tunnel crack information, and the crack information is quantitatively characterized. The specific process of image detection method for tunnel cracks is shown in Figure 2.

## 3. Image Enhancement Algorithm of Multi-Scale Retinex Decomposition Based on Improved Central Filtering

In general, there is no strong light source in the tunnel and auxiliary lighting is needed when collecting the original tunnel crack image. Even if there is a light source, there will be cases where the details of the cracks in the tunnel crack image are not prominent and need to be enhanced. Because the Retinex theory believes that the color of the object is not affected by light [23], the Retinex image model can be used to simulate the human visual perception system. Assuming that U is the collected tunnel image, which is decomposed into two parts, the illumination image Γ and the reflection image E. The collected tunnel image is filtered according to the central surround function to obtain the illumination image, and the reflected image is obtained by logarithmic transformation.

(1) log(U)=log(Γ)+log(E)

(2) E=exp[log(U)−log(Γ)]

To highlight the details and texture structure of the tunnel image, combined with the anisotropy and edge retention ability of bilateral filtering, an improved bilateral filtering method is applied to Retinex decomposition.

The traditional bilateral filtering is

(3) $$\hat{U}(i,j)=\frac{\sum_{(k,l)\in M_{i,j}}w_{s}(k,l)\times w_{r}(k,l)\times U(k,l)}{\sum_{(k,l)\in M_{i,j}}w_{s}(k,l)\times w_{r}(k,l)}$$

where $\hat{U}$ is the filtered image; M(i,j) is the (2N+1)×(2N+1)-space neighborhood set centered on the pixel (i,j); U(k,l) is the pixel value at (k,l) in M(i,j); $w_{s}(k,l)$ is the spatial proximity factor and $w_{s}=\exp\{-[{\left(i-k\right)}^{2}+{\left(j-l\right)}^{2}]/(2\sigma_{d}^{2})\}$; $\sigma_{d}$ is the standard deviation of the spatial domain, which is adaptive to the size of the filtering neighborhood; $w_{r}(k,l)$ is the gray similarity factor and $w_{r}=\exp\{-{\left[U(i,j)-U(k,l)\right]}^{2}/(2\sigma_{s}^{2})\}$; and $\sigma_{s}$ is the standard deviation of the gray domain, which is adaptive to the smooth region and the detail region of the image. The standard deviation of the smooth region is small, and the standard deviation of the detail region is large. Because traditional bilateral filtering ignores the characteristics of the image and the spatial neighborhood, its spatial proximity factor and gray similarity factor have the same weight, which leads to the lack of robustness of the filtering. According to the characteristics of small pixel variance in the smooth region and large pixel variance in the detail region, an improved bilateral filtering model is established by (4) and (5).

(4) $$\hat{U}(i,j)=\frac{\sum_{(k,l)\in M_{i,j}}W(k,l)\times U(k,l)}{\sum_{(k,l)\in M_{i,j}}W(k,l)}$$

(5) $$W=\alpha w_{d}+(1-\alpha )w_{s}$$

where W(k,l) is the weight coefficient of bilateral filtering given by $W(k,l)=\frac{w_{s}(k,l)w_{r}(k,l)}{c}$, and c is a constant. The weight coefficient of bilateral filtering is affected by both the spatial proximity factor and the gray similarity factor. Bilateral filtering not only considers the spatial distance of pixels but also considers the radiation difference in the pixel range domain and has the ability of edge recognition. In (7), α is used to adjust the components of $w_{d}$ and $w_{s}$ in the weight W so as to better maintain the edge and detail structure of the image and give $w_{s}$ greater weight. The improved bilateral filtering is used as the center surrounding function of Retinex to decompose the image, which can effectively separate the illumination image and the reflection image of the image so that the subsequent enhancement of the image can effectively improve the contrast and reduce the distortion of the image [24].

The effect of the improved bilateral filtering depends on the size of its filtering neighborhood. The small neighborhood filtering can retain more details and texture structure, and the large neighborhood filtering has a better smoothing effect. If $r_{1}=5$, $r_{2}=10$, and $r_{3}=15$, they are used to the bilateral filtering radius of the three-scale Retinex decomposition, respectively, and then obtain the small-, medium-, and large-scale illumination images $\Gamma_{1}$, $\Gamma_{2}$, and $\Gamma_{3}$, and reflection images $E_{1}$, $E_{2}$, and $E_{3}$ of the original image. γ correction is performed on the corresponding illumination image to obtain the corrected image $\hat{\Gamma_{l}}$ and $\hat{\Gamma_{l}}={\left(\Gamma_{l}\right)}^{\gamma }(l=1,2,3)$, the mean image is taken from the small-, medium-, and large-scale illumination images and reflection images after γ correction, and the enhanced image is obtained by anti-Retinex transformation, as shown in (6).

(6) $$U_{a}=\exp[\log(\Gamma_{a})+\log(E_{a})]$$

where $\Gamma_{a}$ and $E_{a}$ are the mean images of the small-, medium-, and large-scale illumination images and reflection images, respectively, and $\Gamma_{a}=(\hat{\Gamma_{1}}+\hat{\Gamma_{2}}+\hat{\Gamma_{3}})/3$, $E_{a}=(E_{1}+E_{2}+E_{3})/3$.

**Figure 3:**
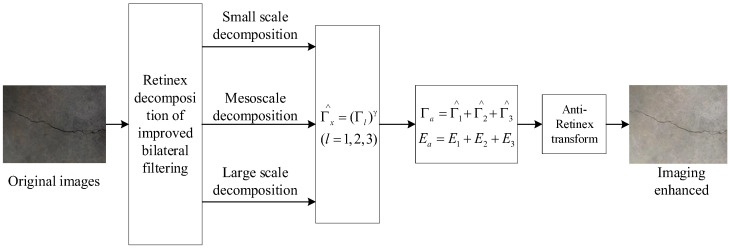
Flowchart of image enhancement algorithm based on improved center filtering multi-scale Retinex decomposition.

The value of γ determines the enhancement effect of the illumination image. When γ<1, the illumination image is subjected to nonlinear lifting transformation. The darker the image area is, the higher the degree of lifting is. The brighter the image area is, the smaller the degree of lifting is, which effectively prevents the bright area from being over-enhanced. According to the characteristics of the image, selecting the appropriate γ value can not only obtain the optimal enhancement effect but also have good universality. According to the mean image of the enhanced multi-scale illumination image and the mean image of the multi-scale reflection image, Retinex reconstruction is performed to obtain the enhanced image, which can reduce the distortion of the image and make the color of the image more natural. The flowchart of this method is shown in Figure 3.

## 4. Crack Segmentation Method Based on Improved VGG19 Network Model

### 4.1. VGG19 Basic Network Model

**Figure 4:**
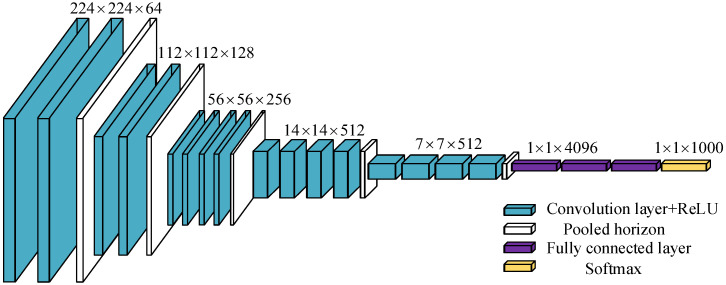
Original structure of VGG19 network.

The VGG network model structure was put forward by the Visual Geometry Group team in 2014 [25,26]. Compared with AlexNet, multiple consecutive 3 × 3 convolution cores replaced the relatively large convolution cores in AlexNet, and the specific VGG19 model structure is shown in Figure 4.

The VGG19 network structure contains five parts. The image input is 224 × 224 × 3 or 224 × 224 × 1 image matrix. The first part of the network structure contains two consecutive convolutional layers. After the first part of the two convolutional layers, the output feature matrix is 224 × 224 × 64, and after passing a layer of 2 × 2 pooling layer, the output feature matrix is 112 × 112 × 64. The second part contains two convolutional layers with 128 3 × 3 convolutional nuclei and a 2 × 2 pooling layer. The output feature matrix of the second part is 56 × 56 × 128, the third part contains four consecutive convolutional layers with 256 3 × 3 convolutional nuclei, and the output feature matrix after a 2 × 2 pooling layer is 28 × 28 × 256. Then, the feature matrix enters the fourth part, which contains four consecutive convolutional layers, each of which has 512 convolutional nuclei, and each convolutional nuclei is 3 × 3. After the pooling operation of a 2 × 2 pooling layer, the output feature matrix is 14 × 14 × 512. Finally, the fifth part also has four consecutive convolutional layers; each convolutional layer has 512 convolutional nuclei, and each convolutional nuclei is 3 × 3. After the convolution operation, the output is 14 × 14 × 512. After a 2 × 2 pooling layer in the fifth part, the output matrix becomes 7 × 7 × 512. After undergoing the fifth part, the output matrix enters the first fully connected layer, and the 7 × 7 × 512 matrix is fully connected after one-dimensional processing. Then, the second and third fully connected layers fully connected the neurons of the former fully connected layer, respectively, and finally, 1000 categories in the classifier softmax were connected with 4096 neurons of the last fully connected layer to classify the images.

### 4.2. Improved VGG19 Network Model

**Figure 5:**
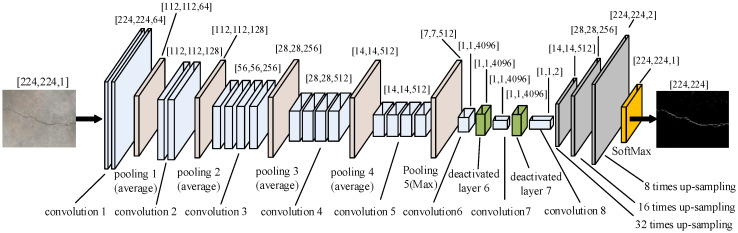
Improved VGG19 network model.

There are three fully connected layers in the VGG19 model, and the number of parameters is large, which requires more memory and consumes more computing resources [27,28]. Therefore, the VGG19 network model is improved to highlight the advantages of the VGG19 network model. The specific improvement is as follows: the downsampling layer after the fifth convolution in the original VGG19 network model is changed to the maximum pooling method, the three fully connected layers in the original VGG19 network are replaced by three convolution layers with the same function, and the image is gradually restored to the original size by upsampling. The upsampling part fuses the overall information and local information in the lining image by adding a specific layer of convolution layer and deconvolution layer, that is, detecting whether there is crack information through the first few convolution layers and then the position of the crack is determined by the deconvolution layer to achieve crack segmentation. The improved VGG19 network structure is shown in Figure 5.

### 4.3. Tunnel Crack Segmentation Method

**Figure 6:**
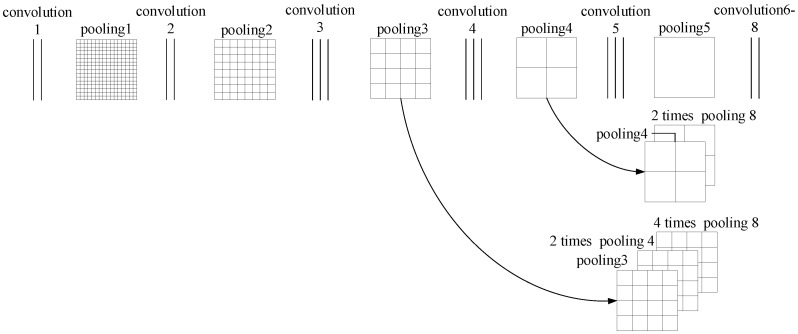
The jump structure of full convolution network.

The size of the feature map is changed to 1/32 of the original map after Group 5 pooling; the fully connected layer in the original VGG19 model is replaced by convolutional layer 6, convolutional layer 7, and convolutional layer 8. In this case, the output heat map is still 1/32 of the original image, although the number of features has changed compared with the feature map output by the pooled layer 5. If the image output by convolutional layer 8 is directly used 32 times upsampling to restore the image to the original size and achieve basic semantic segmentation, but the obtained crack location information is rough, in order to restore the image to the original size and obtain the crack segmentation image with high recognition accuracy, it is necessary to design the skip structure of the full convolutional network [29,30]. In this paper, the skip structure fuses the output results of different pooling layers by upsampling to the same size and then uniformly uses 8 times upsampling to obtain the crack segmentation image with the same size as the input crack image and high recognition accuracy. The jump structure of the full convolution network is shown in Figure 6.

**Figure 7:**
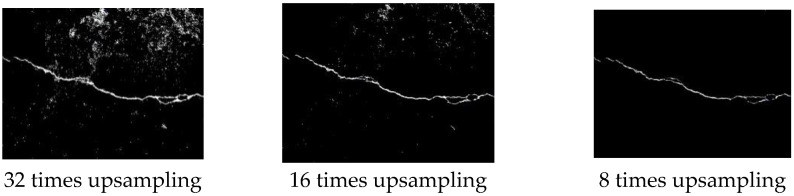
Tunnel crack segmentation images.

The tunnel crack segmentation images obtained by 32 times upsampling, 16 times upsampling, and 8 times upsampling are shown in Figure 7.

## 5. Quantitative Characterization of Cracks

### 5.1. Calculation of Crack’s Length

**Figure 8:**
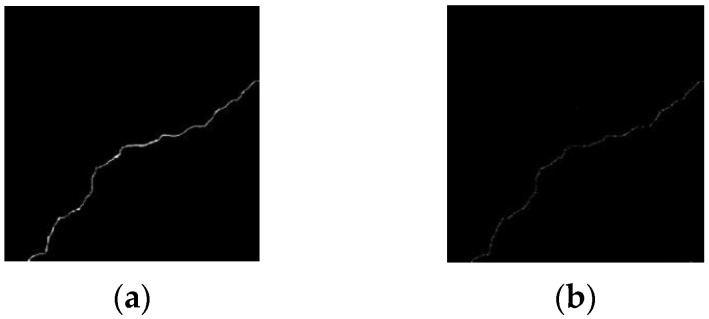
Fracture skeleton extraction effect diagrams. (**a**) Segmentation of fracture binary map by improved VGG19 network and (**b**) fracture skeleton diagram.

The thickness of the tunnel crack is uneven along the trend, and the length of the crack is calculated according to the length of the crack center line. Extract the crack skeleton from the segmented binary image, and the Zhang–Suen fast parallel-thinning method is used to obtain the skeleton map of the single-layer pixel [9]. The crack skeleton extraction results are shown in Figure 8.

On the basis of the fracture skeleton diagram, the length of the crack can be calculated by introducing the Freeman chain code, which describes the curve using the coordinates of the starting point of the curve and the direction code of the boundary point. This paper adopts an eight-connected chain code during the encoding process; the crack endpoint is used as the starting point to encode the cracks, in turn, until the scanning is completed and the chain code representation of a crack is obtained. Suppose that a crack is λ(x,y), the Freeman chain code is used to encode, and then the length of the chain code is calculated; that is, the length of the crack is obtained. The calculation model is as follows:

(7) $$L=\alpha \lambda_{a}+\beta \lambda_{b}$$

where L is the length of the chain code, $\lambda_{a}$ is the number of even chain codes, $\lambda_{b}$ is the number of odd chain codes, and α and β are the corresponding weight parameters, respectively. At the even number chain code, the direction is horizontal or vertical. At this time, the length is the number of pixels, and α=1 is taken. At the odd chain code, the crack direction is ±45°. The segment length is $\sqrt{2}$ times the number of pixels, and $\beta =\sqrt{2}$ is taken.

### 5.2. Calculating Crack Width

**Figure 9:**
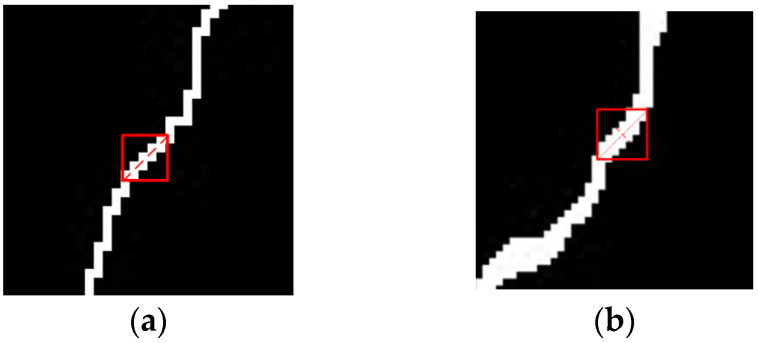
Scan diagrams of crack width. (**a**) Skeleton diagram and (**b**) binary image of original fracture.

Based on the extracted skeleton, the maximum and minimum width of the crack can be obtained by counting the number of normal pixels of the crack at each point on the crack skeleton line. For the local maximum width, the whole crack can be scanned with an n×n sliding window along the crack skeleton trend. Usually, the value of n is between three and five, and the direction of the local straight line is determined by the Freeman chain code value, as shown in Figure 9a. Then, the range of the edges on both sides of the crack is determined along the normal direction of the crack in the original binary image of the crack, as shown in Figure 9b. The length of this dotted line is the local width of the crack. After obtaining the local widths $b_{1},b_{2},\cdots ,b_{n}$, the local maximum crack width $b_{\max}$ of the crack can be obtained by sorting.

## 6. Experimental and Analysis

**Figure 10:**
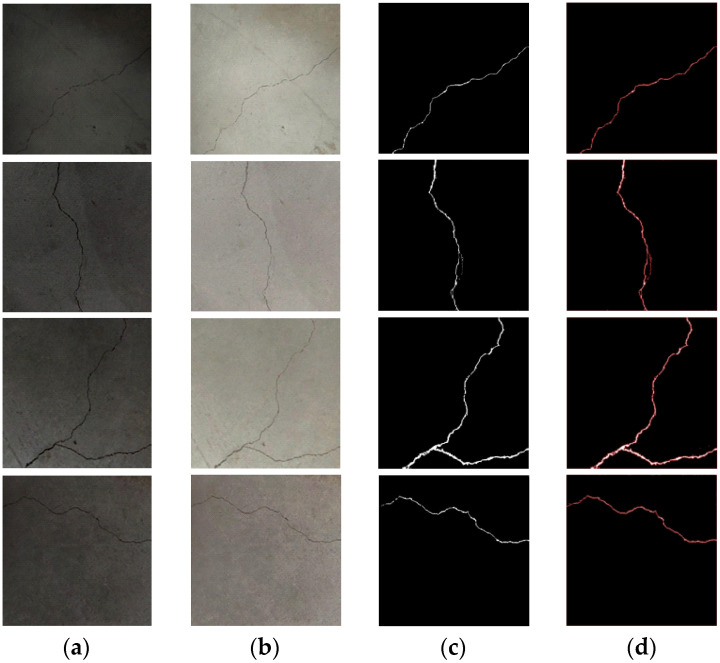
The detection results of the tunnel crack detection method proposed in this paper. (**a**) The 4 original images; (**b**) Retinex enhancement algorithm based on improved central filtering; (**c**) improved VGG19 network segmentation; and (**d**) detection result.

**Figure 11:**
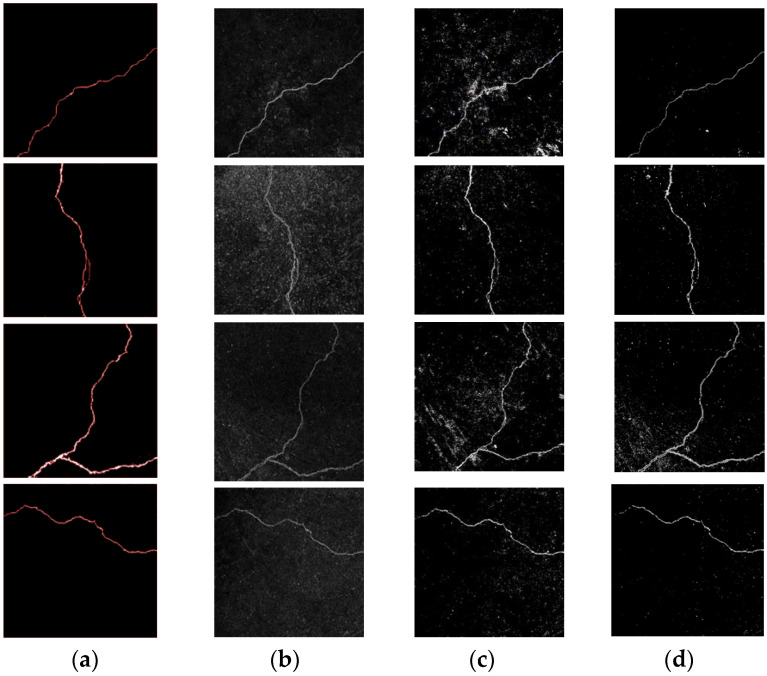
Tunnel crack detection comparison results. (**a**) Detection results by using the algorithm proposed in this paper in Figure 10; (**b**) detection algorithm based on Kirsch and Canny; (**c**) improved OTSU algorithm based on attribute-weighted naive Bayes algorithm; and (**d**) detection algorithm based on ST-YOLO.

In the experiment, 500 original images of tunnel cracks are selected, and 4500 images are obtained after dataset amplification. The improved VGG19 network model is trained in the Windows 10 operating system with an NVIDIA GeForce RTX 3050 laptop GPU, an i5-11400H CPU, and 16GB of memory; the software used is Matlab R2022a and Python3.9, and the deep learning framework used is Tensorflow2.6. In order to verify the reliability and detection accuracy of the proposed method, a comparative test of detection accuracy was carried out. The crack images of different sections of the same tunnel were collected, four original tunnel crack images collected in different sections were randomly selected, and the tunnel crack detection method proposed in this paper was adopted for detection, as shown in Figure 10. The detection results of tunnel cracks were obtained by using the Kirsch and Canny detection algorithms based on Reference [31], the improved OTSU algorithm based on a naive Bayes algorithm based on attribute weighting in Reference [32], and the ST-YOLO detection algorithm based on Reference [33]; Figure 11 shows the comparison results.

**Figure 12:**
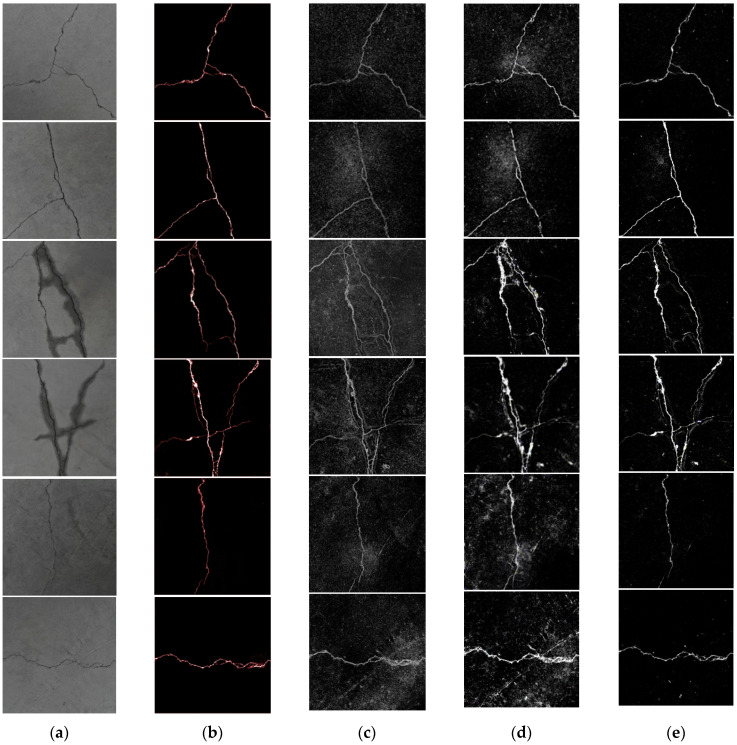
Tunnel crack detection results from different detection methods. (**a**) The 6 original images; (**b**) this article’s detection method; (**c**) detection algorithm based on Kirsch and Canny; (**d**) improved OTSU algorithm based on attribute-weighted naive Bayes algorithm; and (**e**) ST-YOLO detection algorithm.

In order to verify the superiority of the proposed algorithm in this article and detect its robust performance, we select two images of cracks with a simple background, two images of cracks with water seepage, and two images of cracks with dents. The detection algorithm based on Kirsch and Canny, an improved OTSU algorithm based on a naive Bayes algorithm with attribute weighting, and the ST-YOLO detection algorithm were selected to compare the detection results between the proposed algorithm and existing crack detection algorithms, as shown in Figure 12.

According to the tunnel crack detection results of Figure 10, Figure 11 and Figure 12, it can be found that segmentation based on Kirsch and Canny detection algorithms generates a large amount of noise due to water seepage and dents, and the details of tunnel cracks are not prominent, resulting in poor detection results. The detection results of the improved OTSU algorithm based on the attribute-weighted naive Bayes algorithm are also affected by the water seepage and the dents, resulting in noise. Compared with the Kirsch and Canny detection algorithms, the noise generated by the improved OTSU algorithm is relatively small, but the non-crack information in the extracted cracks occupies a large proportion, especially in the case of water seepage in the cracks, the non-crack part in the cracks occupies a larger proportion. For the ST-YOLO detection algorithm, when detecting tunnel cracks, compared with the OTSU algorithm based on the Kirsch and Canny detection algorithm and the improved OTSU algorithm based on attribute-weighted naive Bayes algorithm, the noise is less, but the image will also appear in the phenomenon of misidentification. The method proposed in this paper can achieve ideal detection results, whether it is to detect tunnel cracks with a relatively simple background or to detect tunnel cracks under water seepage conditions. Therefore, the method proposed in this paper has better performance and more robustness.

In order to further verify the superiority of the Retinex enhancement algorithm with improved center filter and the improved VGG19 network model proposed in this paper in detecting tunnel cracks, The proposed method was compared with the detection time of the Kirsch and Canny detection algorithms based on Reference [31], the improved OTSU algorithm based on a naive Bayes algorithm based on attribute weighting based on Reference [32], and the ST-YOLO detection algorithm based on Reference [33]. When the deep learning network model was trained in this paper, the InitialLearnRate was set to 0.001, MaxEpochs to 8, MiniBatchSize to 10, the weight attenuation coefficient and momentum parameters were set to 0.0005 and 0.9, respectively, and the maximum number of iterations was set to 1000. Under the same training parameter conditions, the crack detection performance pairs of various methods are shown in Table 1.

It can be seen from Table 1 that the improved OTSU algorithm based on the attribute-weighted naive Bayes algorithm in Reference [32] has great advantages in detection time, but the effect of defect detection is not ideal. The accuracy rate is only 70.9%, and the loss rate is as high as 27.2%. Because the traditional machine learning method relies heavily on prior knowledge in image feature extraction, and it is highly dependent on data, which requires a large amount of data to train the model, the crack detection method based on Kirsch and Canny in Reference [31] can effectively improve the accuracy of detection and greatly reduce the loss rate compared with the tunnel crack detection method based on OTSU algorithm improved by attribute-weighted naive Bayes algorithm in Reference [32]. However, Reference [31] mainly uses the combination of the Kirsch operator and the Canny operator to realize the edge detection of cracks; thus, it is difficult to effectively deal with the problem of uneven illumination in tunnel crack images, and the detection accuracy is still poor. The tunnel detection method based on ST-YOLO in Reference [33] is compared with the detection method based on Kirsch and Canny in Reference [31] and the improved OTSU algorithm based on the attribute-weighted naive Bayes algorithm in Reference [32]. Although the accuracy of detection is higher, the detection time is longer, and the accuracy of detection is still lower than the improved VGG19 network model established in this paper. The accuracy of the improved VGG19 network model detection in this paper is as high as 95.93%, which is 8.33% higher than the ST-YOLO detection algorithm based on Reference [33]. The reason is that the improved VGG19 network model replaces the three fully connected layers in the original VGG19 network with three convolutional layers with the same function and uses the upsampling method to gradually restore the image to the original size, which improves the detection rate and reduces the detection time.

The four original images in Figure 10 and the six original images in Figure 12 are processed by different detection methods. The improved VGG19 network proposed in this paper is used to segment tunnel cracks. The length and width of cracks are measured on the basis of crack skeleton extraction. In addition, the length and width of cracks in tunnel crack images processed by the Kirsch and Canny detection algorithm, OTSU algorithm improved by attribute-weighted naive Bayes algorithm, ST-YOLO algorithm, and manual measurement method are measured. The detection results of the crack lengths are shown in Table 2, and the results of crack width detection are shown in Table 3.

It can be seen from Table 2 and Table 3 that the absolute error of the tunnel crack detection method based on the improved OTSU algorithm based on the attribute-weighted naive Bayes algorithm in Reference [32] is significantly higher than that of the other three detection methods when measuring the length and width of tunnel cracks. The reason is that when the method is used to measure the length and width of tunnel cracks, it is affected by crack seepage and dents, resulting in a large deviation between the measurement results and manual detection. The crack detection method based on Kirsch and Canny in Reference [31] is compared with the tunnel crack detection method based on the improved OTSU algorithm based on the attribute-weighted naive Bayes algorithm in Reference [32]. Although the measurement error is relatively small when measuring the length and width of tunnel cracks, the error fluctuation of the crack detection method based on Kirsch and Canny in Reference [31] is large. The reason is that for some cracks whose edge details are not obvious, this method cannot detect them well. The tunnel detection method based on ST-YOLO in Reference [33], when measuring the length and width of tunnel cracks, compared with the crack detection method based on Kirsch and Canny in Reference [31] and the tunnel crack detection method based on attribute-weighted naive Bayes algorithm improved OTSU algorithm in Reference [32], although the absolute error is relatively small, when measuring the length and width of tunnel cracks, when water seepage and dents appear in tunnel cracks, the deviation from the manual measurement results is also large, and the maximum deviation in crack length is about 10 mm. The maximum deviation in crack width is about 1.3 mm. The results show that the absolute error of the detection method proposed in this paper is significantly lower than that of the first three detection methods when measuring the length and width of tunnel cracks. The maximum deviation in crack length is about 5 mm, and the maximum deviation in crack width is about 0.8 mm.

## 7. Conclusions

Due to insufficient illumination, when collecting tunnel cracks, the overall brightness of the collected tunnel crack images is not uniform, and the imaging quality is deviated, which is not conducive to the identification of cracks. In order to offset the lack of illumination and the inconsistency of image brightness distribution, this paper proposes an image enhancement algorithm based on improved center filtering multi-scale Retinex decomposition and an improved VGG19 tunnel crack detection method to achieve efficient segmentation of cracks. The experimental results show that the accuracy of the crack detection results of the proposed method is 12.03%, 25.03%, and 8.33% higher than that of References [31,32,33], respectively, and the loss rate is reduced by 4.7%, 18.5%, and 2.7%, respectively. In summary, the method proposed in this paper can greatly reduce the influence of the surrounding environment and the illumination of the shooting and better remove the interference of noise on the crack segmentation, making the crack segmentation details more accurate. The accuracy of the proposed detection method for crack segmentation is much higher than that of the other detection methods in the comparisons and meets the real-time requirements. The method proposed in this paper is to collect tunnel cracks in the way of a plane-array camera and a fill light and introduce multi-scale Retinex decomposition of bilateral filtering to enhance the image. According to the improved VGG19 network model, the efficient segmentation of tunnel cracks and the quantitative characterization of cracks can be realized. These works can not only detect irregular cracks in the tunnel but also detect irregular cracks in the tunnel. Therefore, the method in this paper is not only applicable to concrete structures but also to crack detection in other environments, such as the crack detection of steel frame structures and tunnel bodies composed of other materials. At the same time, it can also detect the pavement of high-speed roads and airstrips. The method has a very broad application prospect.

## Figures and Tables

**Table 1:** Comparison of crack detection performance of various methods.

Crack Detection Method	Detection Time/s	Detection Results	
		Accuracy	Loss Rate
The improved VGG19 network model in this paper	309.9	95.93%	8.7%
Detection algorithm based on Kirsch and Canny	431.7	83.9%	13.4%
Improved OTSU algorithm based on attribute-weighted naive Bayes algorithm	281.9	70.9%	27.2%
Detection algorithm based on ST-YOLO	476.4	87.6%	11.4%

**Table 2:** Crack length test results.

NO.	Manual Measurement	Measured Values in This Paper	Detection Method Based on Kirsch and Canny	Improved OTSU Algorithm Based on Attribute-Weighted Naive Bayes Algorithm	ST-YOLO Detection Algorithm
1	368	372	375	379	373
2	385	382	379	380	381
3	453	457	445	462	459
4	342	345	344	348	346
5	495	491	488	503	489
6	421	425	416	428	424
7	436	438	426	447	441
8	429	434	437	441	439
9	397	394	401	403	391
10	391	389	395	398	387

**Table 3:** Crack width test results.

NO.	Manual Measurement	Measured Values in This Article	Detection Method Based on Kirsch and Canny	Improved OTSU Algorithm Based on Attribute-Weighted Naive Bayes Algorithm	ST-YOLO Detection Algorithm
1	6.9	7.1	6.6	7.5	7.3
2	8.7	9.2	8.1	9.4	9.3
3	13.5	13.7	12.9	14.3	13.9
4	7.2	7.5	6.5	7.9	7.6
5	9.6	10.2	8.7	10.8	9.4
6	10.8	10.4	9.8	11.9	11.5
7	10.1	10.9	11.7	12.2	11.4
8	11.6	11.8	10.5	13.4	12.7
9	6.4	6.1	7.1	7.5	6.9
10	8.3	8.5	8.9	9.2	8.6

## Data Availability

The data that support the findings of this study are available from the corresponding author upon reasonable request.
